# Magnetic Resonance Imaging in patients with ICDs and Pacemakers

**Published:** 2005-07-01

**Authors:** Prashant Nair, Ariel Roguin

**Affiliations:** Department of Cardiology, Rambam Medical Center, B. Rappaport - Faculty of Medicine, Technion - Israel Institute of Technology, Haifa, Israel

**Keywords:** Imaging, MRI, pacemaker, ICD

## Abstract

Magnetic resonance (MR) imaging has unparalleled soft-tissue imaging capabilities. The presence of devices such as pacemakers and implantable cardioverter/defibrillators (ICDs), however, is historically considered a contraindication to MR imaging. These devices are now smaller, with less magnetic material and improved electromagnetic interference protection. This review summarizes the potential hazards of the device-MR environment interaction, and presents updated information regarding in-vivo and in-vitro experiments. Recent reports on patients with implantable pacemakers and ICDs who underwent MR scan shows that under certain conditions patients with these implanted systems may benefit from this imaging modality. The data presented suggests that certain modern pacemaker and ICD systems may indeed be MR safe. This may have major clinical implications on current imaging practice.

## Introduction

Magnetic Resonance (MR) imaging is a diagnostic technique used to obtain high quality images of the human body. The structure and abundance of water in the different tissues of the human body is the key to clinical MR imaging. The basic concept of MR is the absorption or emission of electromagnetic energy by atomic nuclei in a static magnetic field after excitation by a radiofrequency (RF) pulse [1]. A powerful magnet generates a magnetic field roughly 10,000 times stronger than the natural background magnetism from the earth. Various types of clinical MR systems currently use the superconductive magnet which utilizes 0.5 Tesla to 3.0 Tesla.

Unlike conventional radiography and computed tomographic imaging, which make use of potentially harmful radiation (X-rays), MR imaging has many advantages, including its nonionizing nature and the unparallel ability to discriminate different soft tissues without contrast media. MR imaging has now become the image modality of choice for imaging the brain, spine, musculoskeletal system, head and neck, complex pediatric heart malformations and other tissue structures [2].

More recently, MR imaging has been applied successfully to assess myocardial structure, wall motion, perfusion and viability. The number of MR scans performed annually has increased dramatically over the past few years [3-6].

## The Growing Problem

**Figure 1:**
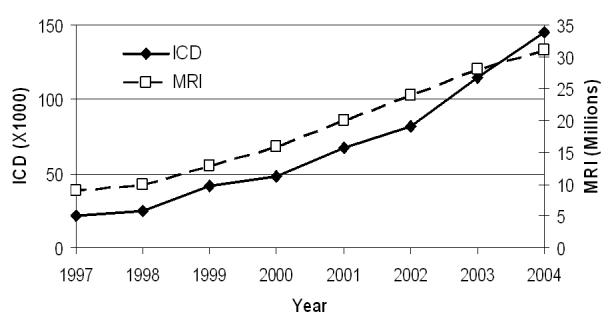
Number of annual MR scans and of newly implanted ICDs in the USA

Parallel to the growth and evolution of the MR field, is the burgeoning number of patients benefiting from implantable cardiac systems including pacemakers and implantable cardioverter-defibrillatos (ICDs) (Figure 1). With new indications for heart failure, innovative device features and expanded medical coverage; this trend is likely to continue its trajectory. The combination of these two growing phenomena results in an estimated 50-75% probability of a patient being indicated for an MR study over the lifetime of their device creating an estimated 200,000 device patients who were denied the MR scan and more in the future [7,8].

Given the rapid expansion of technology in the fields of both MR imaging and device arrhythmia management, there is increasing interest in the issue of implantable device safety in the MR environment. Currently no implantable cardiac device has Food and Drug Agency (FDA) approval for use in the MR environment and “Do not use magnetic resonance imaging on patients who have an implanted device” appears on product labels [8]. The current state of affairs significantly limits the performance of MR imaging on device patients. With a better understanding of the hazards of performing MR scans on device patients as well as the development of MR safe devices, we may soon enter an era where the ability of this imaging modality may be more widely used to assist in the appropriate diagnosis of patients with devices. Not only for heart imaging but mainly for brain, spine, and joints as knees and shoulder [1-7].

## Hazards and Safety concerns

Permanent cardiac pacemakers have represented a contraindication to MR imaging. Strong static, gradient and radiofrequency fields used for MR image creation are thought to be detrimental to pacemaker function and cause harm to patients undergoing MR examinations. The multiple potential adverse interactions between pacemakers and MR imaging [1,2,9-12] (Table 1) include heating, rapid atrial pacing, pacing at multiples of the RF pulse and associated rapid ventricular pacing, reed switch malfunction, asynchronous pacing, inhibition of pacing output, alteration of programming with potential damage to the pacemaker circuitry or movement of the device and the potential thermal injury at the lead tip.

Supporting the current practice comes from several reported lethal consequences of MR imaging in patients with implanted pacemakers [13-15]. During the late 1980s incidentally 10 deaths have been attributed to MR procedures in patients with pacemakers. However these fatalities were poorly characterized and no electrocardiographic data were available. Most importantly no deaths have been reported during physician supervised MR procedures in the last decade.

Despite the above-mentioned concerns, the effects of MR on cardiac pacemakers remain controversial. Most of the previous studies that prohibit MR in pacemaker patients were based on in vitro and animal model data in the 1980’s using older pacemaker and lead technology. During the last decade, anecdotal reports describe a small series [16-21] of pacemaker patients who have safely undergone magnetic resonance scanning (Table 2). Advance in device technology drove extensive and seminal in-vitro and animal studies of the pacemaker and ICD systems interaction with the MR, and in recent years, several groups scanned safely larger number of patients.

## In-Vitro and Animal Studies

The potential hazardous effects of MR imaging in patients with cardiac pacemakers have been studied since 1983. Pavlicek and colleagues were the first to report the effects of MR imaging on pacemaker function [11]. They showed that RF fields present in an MR unit could possibly inhibit demand pacemakers and time-varying magnetic fields could generate pulse amplitudes to mimic cardiac activity. The threshold for initiating the asynchronous mode of a pacemaker was reported to be as low as 17 Gauss (1 Gauss = 10-4 Tesla). The possibility of altering pacemaker parameters was presented as a serious limitation of MR imaging. Fetter et al [12]. showed that pacemakers reverted from the demand to the asynchronous mode within the magnetic field of the scanner (0.15 Tesla), but microscopic testing showed no evidence of reed switch sticking or magnetizing, or damage to other discrete pacemaker components. Other investigators studied the feasibility of dual-chamber pacing systems in the MR environment. Erlebacher et al. tested different DDD pacemakers in a saline phantom, and showed that during scanning at 0.5 Tesla, all units malfunctioned due to RF interference which caused total inhibition of atrial and ventricular output, or resulted in atrial pacing at very high rates [9]. The potential for rapid cardiac stimulation during MR was also reported in animal studies [2]. Lauck et al. investigated the performance of different stimulation modes (VVI, VVIR, VOO, DDD, DDDR and DOO) during MR scan at 0.5 Tesla [23]. Reversible activation of the reed switch with consecutive asynchronous stimulation was observed in all pacemakers. Pacemakers in the asynchronous mode were not affected with regard to stimulation rate and capture during scanning. In contrast, pacemakers with automatic mode switching to demand pacing or programmed inactivation of the reed switch were triggered in the dual chamber mode and were inhibited in the single chamber mode. Thus, the investigators recommended programming into the asynchronous mode prior to scanning, and in those without permanent pacemaker dependency, complete inactivation of the system, if possible.

The effects of more powerful MR scanners (i.e., 1.5 Tesla) on cardiac pacemakers were initially reported by Hayes et al [24]. In-vivo evaluation of different single and dual chamber pacemakers showed reversion into asynchronous mode and transient reed switch inhibition. Seven of the eight pulse generators paced rapidly when exposed to the RF signal associated with a marked decrease in blood pressure. Stimulation cycle length was 200 ms (300 beats/min) corresponding to the frequency of pulsing. It was proposed that rapid pacing was the result of an "antenna" effect that couples the RF energy back into the pacemaker output circuits.

More recently, Achenbach et al [25] showed in a phantom study on 11 pacemakers and 25 leads that no pacemaker malfunction was observed in asynchronous pacing mode (VOO/DOO), whereas inhibition and rapid pacing were observed during spin-echo imaging if the pacemakers were set to VVI or DDD mode. The authors suggested that rapid pacing was caused by induction of currents above sensing threshold in the atrial lead and consequent triggering of ventricular stimulation. Direct interference with the pacemaker electronics seemed to be an unlikely explanation, because the rapid pacing rate was always equal to the programmed frequency limit.

Importantly, most of the above were reports of earlier generation pacemakers. Recent reports testing improved technology devices found no functional issues in most pacemakers exposed to prolonged MR scan [26,27].

Measuring lead heat in the MR environment is technically difficult and depends on the methods used; explaining why several group report different results. Heating effect of pacemaker leads was investigated by Achenbach et al [25]. Continuous registration of the temperature at the lead tip using an optical temperature sensor showed a maximal temperature increase of 63.1°C during 90 seconds of scanning. In seven electrodes, the temperature increase exceeded 15°C. Luechinger et al [28] used pacemaker leads with additional thermocouple wires as temperature sensors implanted in nine animals to measure heating. They recorded temperature increases of up to 20°C were during MR imaging of the heart. However, they found only minor stimulation threshold changes and no pathology and histology heat-induced damage.

Roguin et al [26] found in-vitro, maximal heating of 7°C. In vivo, when the leads were inserted into the right ventricle, there was almost no rise in temperature. This was probably due to the blood flow and heat dissipation. More importantly, in a chronic animal model; of 15 dogs who had ICD leads implanted and all scanned during prolonged (3-4 hours) MR scans, including high energy protocols - revealed no heat induced injury. No heat and injury was recently also reported by a Swiss group [27].

With regard to force and torque, several studies found that in pacemakers the force in negligible around 10 grams while with ICD it depends on the year it was manufactured and the amount of ferromagnetic material. Older devices exert significant forces of 400-500 grams however most newer ICDs exert around 100 grams [26-29].

## Human Studies

### Reports using older technology

In the earliest years of MR, using older generation pacemakers devices, as mentioned above there were few anecdotal reports of unexpected deaths in patients undergoing MR imaging [13-15]. In one case, the patient had no escape ventricular rhythm and apparently died due to systole. Another patient developed ventricular fibrillation during the imaging procedure that was not recognized immediately because ECG monitoring was not used [30]. On the other hand, there are also reports of pacemaker patients who underwent MR imaging safely (Table 2).

Therefore, differences exist among clinicians regarding the perceived safety of scanning paced patients. In patients who underwent MR imaging of the head, no pacemaker malfunction was observed with the pacemaker turned off or programmed to an asynchronous pacing mode prior to MR exposure [16-20]. In another study on five patients with pacemakers, Gimbel et al. reported normal pacemaker performance in four patients during MR (0.35 and 1.5 Tesla) [18]. One patient had a pause of approximately two seconds in duration near the completion of MR scan, the cause of which could not be determined. This occurred in a pacemaker dependent patient with a unipolar dual chamber device programmed to DOO mode. No rapid cardiac pacing occurred and no patient reported a torque or heating sensation. Fontaine et al. reported a case of rapid cardiac pacing during MR imaging (1.5 Tesla) in a patient with a dual chamber pacemaker [19]. The patient developed an irregular ventricular rhythm during RF pulsing which terminated with the cessation of RF pulsing. MR at 0.5 Tesla was shown to have no influence on atrial and ventricular stimulation thresholds, P and R wave amplitudes, electrode impedance, battery voltage, current, and impedance measurements in patients with implanted pacemakers.

### Reports using present technology

Vahlhaus et al [31] reported their experience using 0.5 Tesla MR system on 34 MR examinations in 32 patients with implanted pacemakers and concluded that MR imaging at 0.5 Tesla does not cause irreversible changes in patients' pacemaker systems. Lead impedance and sensing and stimulation thresholds did not change after MR imaging. Battery voltage decreased immediately after MR imaging and recovered 3 months later.

In a recent study [32], the largest human report so far, 54 non dependent permanent pacemaker patients underwent 64 MR examinations at 1.5 Tesla. Only 9.4 % of the leads underwent significant threshold changes and were easily addressed with subtle programming changes. Patient's symptoms and electrographic changes were mild and transient and did not warrant cessation of MR scan. The authors concluded that the “performance of unrestricted MR procedures using a 1.5-Tesla MR system was found to have an acceptable safety profile”. They cautioned, however, that the wide variety of pacing systems and electromagnetic fields used in MR procedures implied that the “absolute safety of pacemaker and MR interactions cannot be assured”.

Schmiedel et al [27] from Bonn, Germany, tested the translational forces and temperature increase (max<2.98°C) that were in a range which does not represent a safety concern from a biophysical point of view. They reported their experience with, 63 MR imaging examinations at 1.5 Tesla in 45 patients with implanted pacemakers. Prior to MR the devices were re-programmed in an asynchronous mode. The maximum Specific absorption rate (SAR) of MR-sequences was limited to 1.2 W/kg. Continuous monitoring of ECG and pulse oximetry was performed during MR imaging. No changes to the programmed parameters of the PM or damage of PM components were observed neither In-vitro (n = 0/24) nor In-vivo (n = 0/63). All patient studies (n = 63/63) could be completed without any complications. Atrial and ventricular stimulation thresholds did not change significantly immediately post-MR imaging nor in the 3 months follow-up. They concluded “MR of the brain at 1.5 Tesla can be safely performed in carefully selected clinical circumstances when appropriate strategies are used (re-programming the device to an asynchronous mode, continuous monitoring of ECG and pulse oximetry, limiting the SAR value of the MR sequences, cardiological stand-by). Based on these studies, implanted pacemaker should not longer be regarded as an absolute contraindication for MR scan at 1.5 Tesla”.

A similar prospective study evaluated the risks of 2.0 T-MR in patients with pacemakers and concluded that MR imaging in patients implanted with the specific device and leads evaluated was safe however cautioned that the results may not be applicable to all MR or pacemaker systems and suggested limited MR exposure to pacemaker patients until larger trials have been conducted (Roguin, personal communication).

### Imaging in Pacemaker Dependent Patients

Little has been presented regarding MR imaging of pacemaker dependent patients. As part of a series of five patients, Gimbel et al [33] reported one pacemaker dependent patient who safely underwent cranial MR imaging. Safe inadvertent scanning of pacemaker dependent patient also has been performed but not published yet [Roguin, personal communication]. The results suggest that pacemaker dependent patients might also be offered MR if careful patient monitoring and pacemaker reprogramming is performed in concert with use of a transmit receive coil (in cranial scans) and implementation of specific MR sequences designed to limit power deposition over the device. A larger series of pacemaker dependent patients need to be evaluated before a benign outcome can be made.

### Safety Issues in Patients with Retained Pacing Leads

Many patients have endocardial pacemaker leads left in place after pulse generator removal. The safety of MR in patients with retained endocardial pacemaker wires has not been systematically investigated to date. However, due to the potential threat that they may act as “antennas” with significant heating - we feel it is not recommended to scan those patients.

Temporary pacing wires, usually made of stainless steel, are sutured to the epicardial surface of the heart over the right ventricle and right atrium after cardiac surgery, and connected to an external pacemaker if the patient develops bradycardia or atrioventricular block. Theoretical calculations using a circuit formed by epicardial pacing wires showed induction of currents up to 80µA by the beating heart in a magnetic field strength of 1.5 Tesla [34]. Hartnell et al [35] investigated the safety of 1 or 1.5 Tesla MR systems operating with conventional pulse sequences in 51 patients with retained epicardial pacing wires, cut short at the skin, after cardiac surgery. None of the patients reported symptoms suggesting arrhythmia or other cardiac dysfunction during MR imaging, and there were no changes from the baseline ECG rhythms. Therefore, retained epicardial wires do not seem to present a hazard to patients in the MR environment. However, this conclusion applies mostly to non-cardiac MR examinations [36].

### Imaging in Patients with Implantable Cardioverter Defibrillators (ICD)

Although different as in the presence of large capacitors and larger batteries that may cause higher magnetic forces., pacemakers and ICDs share similar components and thus, to some extent, their response to the electromagnetic interference (EMI) present during MR scanning might be expected to be similar [1,8,14]. Despite dramatic reduction in size and weight, new generation ICDs have 10 times higher magnetic torque. When tested this was found to be around 100gram/cm as compared to 10 gram/cm for pacemakers. Of note, for older ICDs (late 1990 models) the torque was >300 gram/cm [26,27]. The implanted device has a fibrotic envelope around it several weeks after implantation. Forces less than 200 gram will not be felt by the patients [26]. ICD devices may falsely detect the MR RF noise as ventricular tachyarrhythmia and subsequently deliver antitachycardia pacing, cardioversion or defibrillation therapies. In addition, magnetic fields may prevent detection of ventricular tachycardia or fibrillation. The heating problem of ICD leads can be expected to be the same as in case of pacemaker leads.

Despite many pacemaker patients having reportedly undergone MR imaging using a variety of strategies to allow safe MR scanning, relatively very little has been reported regarding ICD patients undergoing deliberate MR. An abstract and two case reports [37-39] have described the ill effects of inadvertent MR imaging of ICD patients. Interestingly the same devices were tested by Roguin et al, and the same findings were found - unable to interrogate these older generation ICDs [26]. One case report of an ICD patient inadvertently undergoing MR imaging noted a substantial rise in pacing thresholds subsequent to the MR exposure [40]. Concerns over a possible rise in defibrillation test (DFT) was recently answered by, a recent preliminary report of ICD patients undergoing MR that showed greater than the 10 J safety margin of safety during post MR imaging DFT testing [32]. No thermal injury was found by Roguin et al, in 15 dogs that underwent prolonged MR scans 4 weeks after ICD implantation [26].

Recently deliberate scanning of ICD patients was reported. Wollmann et al [41] report on a patient with an ICD who intentionally underwent MR imaging of a malignant brain tumor. The ICD was inactivated by programming the VT-detection and VT/VF-therapy status off. The patient came through the protocol safely and without any difficulty or discomfort. There was no arrhythmic event. MR imaging affected neither programmed data nor the function of the ICD system. Roguin et al [42], based on their in-vitro and in-vivo results, implanted an ICD that was found to be safe, in a young patient with ventricular arrhythmia and suspected ARVD. To confirm the diagnosis a follow up MR scan was advised. So the patient underwent intentional MR imaging 6 weeks after implantation. The scan was safe and most of the MR images were of high quality.

Gimbel et al [43] reported their experience on seven patients who underwent eight MR imaging scans at 1.5 Tesla. Post-MR scan, all devices demonstrated no change in pacing, sensing, impedances, charge times, or battery status. None of the patients had any discomfort. They concluded that scanning of ICD patients might be performed if appropriate reprogramming and monitoring is implemented. Several patients with the newer biventricular ICD systems, were safely scanned [Roguin, personal communication].

The rapidly accumulating number of safely scanned patients with pacemakers directs one to surmise that we might also safely scan patients with ICDs if similar strategies that had allowed safe MR in pacemaker patients were applied to ICD patients. Some investigators [26-44] have suggested that “modern” devices are less prone to the effects of MR and because of better built in EMI protection circuitry. Disabling the tachy-arrhythmia detection and therapy is one strategy which has been recommended. Programming to therapy off avoids delivering therapy as a result of interpretation of noise as tachyarrythmia [26]. In the study carried out by Gimbel et al [43], simple strategies that had allowed patients with pacemakers to safely undergo MR imaging were applied to several ICD patients.

## Image quality

Though lot of studies have been done to determine whether the devices are fully MR imaging-compatible (function appropriately and without significant image distortion) very little is known about the fact whether these device are MR-safe (function appropriately in the MR environment but distort the image) or may not be usable in an MR scanner [44,45]. Image artifacts and RF noise can be caused by the presence implanted devices in the MR environment which are in or near the imaging field of view (such as implants or surface electrodes). These materials produce their own characteristic static magnetic field that can perturb the relationship between position and frequency essential to accurate image reconstruction. If the object has a magnetic susceptibility that is significantly different from that of tissue, distortion will result. Primary concerns with image artifact and noise include the production of a void where anatomical information is needed as well as the production of artifacts that may be misdiagnosed as pathology. Schueler et al [45] have offered an assessment of image quality according to four criteria: geometric distortion; susceptibility-induced artifact; warping artifact; and bending, warping, or obliteration of image contours.

Most artifacts from pacemakers and leads result in local image distortion, signal voids, or increased noise. In an in vitro study [26] in a dog model using the clinical scanning protocols this issue was addressed and image distortion was analyzed by measuring the area where there was a void in the MR image. The distortion was analyzed at the level of the heart and at the level of the device. Artifacts due to the devices and leads were observed in some MR sequences and less in others. Most of the distortion was dependent on scan protocol and image plane: Fast Spin Echo and FIESTA sequences had significant distortion.  Fast Gradient Recalled Echo, Tagging and FSPGR sequences, however, yielded good images. Larger artifacts were observed in image planes roughly parallel to the planes defined by the device itself. Most distortion was at a distance of 10-15 cm around the device generator. The authors concluded that image distortion was dependent on the imaging plane and protocol used. Most image distortion was in the area adjacent to the device generator. Therefore, organs visualization beyond this distance, such as knees, lower spine, liver or brain, will not be affected by the presence of the pacemaker or ICD.

## Summary

Most researchers in the field agree that although several hundreds of patients with implanted devices underwent safe MR scan - this does not conclude that MR imaging in patients with pacemakers or ICDs is indeed safe. According to the FDA’s definition, today’s ICDs are not “MR Safe” nor “MR compatible”. Because of the small size of the series and limited to few manufacturers one cannot conclude that MR imaging can deliberately be performed routinely in Pacemaker/ICD patients without risk. Further study is required to better understand the interaction between pacemakers and ICD systems and the effects of MR imaging.

Ultimately, a truly “MR safe” ICD system will need to be developed. Nevertheless, one should be encouraged, and like the experience with pacemakers, the initial encouraging reports of MR imaging in ICD patients reported only a small series.

Like pacemaker patients, ICD patients should not be considered for MR scan unless appropriate precautions are undertaken and then only when other avenues to acquire clinically relevant imaging data have been explored. Should MR imaging be considered in a patient with an implantable device, appropriate consultation with a qualified cardiologist or electrophysiologist is strongly advised so that patients can be adequately screened and correctable physiologic abnormalities (e.g. hypoxia, electrolyte abnormalities, etc.) can be addressed prior to MR imaging. The patient must not be pacemaker/ICD dependent. The clinician needs to document in the chart, that MR imaging is crucial to the management of the patient. No other imaging can be expected to provide an alternative or has been unsuccessfully tried. The patient or family will sign a preprinted consent form explaining potential adverse reactions, invalidation of warranty, heating leads, rapid pacing, alteration of programming, damage of circuitry requiring replacement and movement of the device.

In 2004, the American College of Radiology (ACR) issued an update of its 2001 MR Safe Practices Guidelines [8] in which they reiterate their recommendation that implantable devices should be a contraindication for MR imaging. They added, however, that decisions for exceptions should be made on a case-by-case basis and that all MR scans should be performed under the guidance of both an experienced radiologist and cardiologist/ electrophysiologist, but noted that “the expertise necessary to safely do so is exceedingly rare throughout the MR industry today”. Thoughtful pre-MR reprogramming, careful monitoring during MR scan and thorough follow-up must be performed in any device patient considered for MR imaging. Finally a physician knowledgeable in device therapy and programming should be present throughout the entire scanning event (Table 3). The authors of the 1.5-T pacemaker study [32] agree with the ACR guidelines that continuous monitoring is needed and that properly trained personnel and physicians with expertise in adjusting device programming interrogate the devices before and after the scan. Adherence to these practice guidelines may ensure the implantable cardiac device patient can safely undergo MR imaging (Table 4). As suggested by the aforementioned studies, it may be an appropriate time to reconsider whether the presence of an implantable cardiac device should be an absolute contraindication to MR imaging.

## Figures and Tables

**Table 1:** Potential effects of MR imaging on pacemaker and ICD systems

1. Static Magnetic Field	Mechanical forces on ferromagnetic components
	Unpredictable magnetic sensor activation, Reed-switch closure
	Changes in electrocardiograms

2. Modulated Radio Frequency (RF) Field	Heating of cardiac tissue adjacent to lead electrodes
	Possible induction of life-threatening arrhythmias
	Pacemaker reprogramming or reset
	RF interactions with the device (over- and under-sensing)

3. Gradient Magnetic Field	Possible induction of life-threatening arrhythmias
	Induced voltages on leads cause over- and under-sensing

4. Combined Field Effects	Alteration of device function due to EMI
	Mechanical forces (vibration)
	Electronic reset of device

**Table 2:**
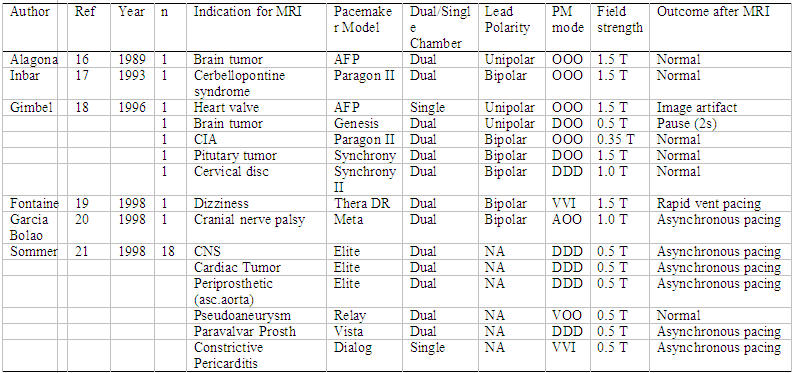
Published reports describing the non-lethal consequences of magnetic resonance imaging n pacemaker patients (n=number of patients studied; n.a.=data not available; PM=pacemaker;T=Tesla)

**Table 3:**
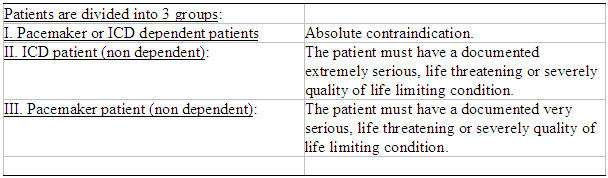
Magnetic Resonance Imaging and Pacemakers: Safety Concerns and Guidelines

* Due to higher degree of interaction between MRI and ICD, the threshold for imaging is higher than for pacemakers.

**Table 4:**
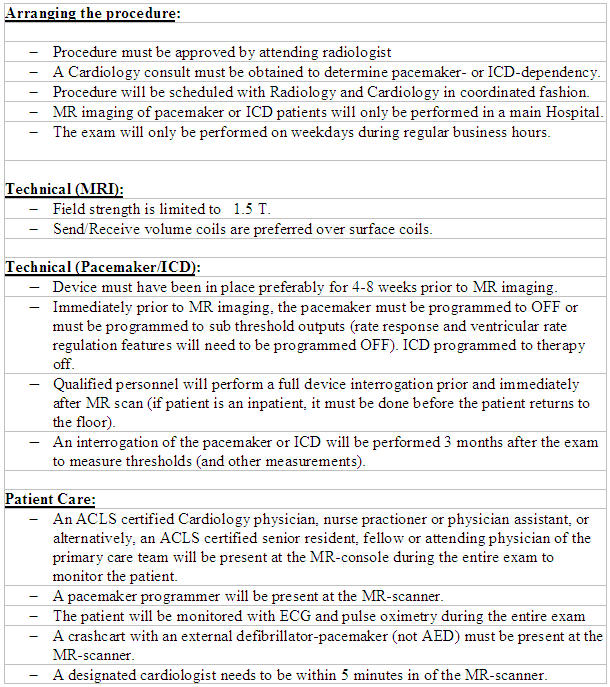
Technical aspects in MR imaging of patients with pacemakers/ICDs
